# Sequential *RAS* mutations evaluation in cell-free DNA of patients with tissue *RAS* wild-type metastatic colorectal cancer: the PERSEIDA (Cohort 2) study

**DOI:** 10.1007/s12094-024-03487-4

**Published:** 2024-04-20

**Authors:** Manuel Valladares-Ayerbes, Maria José Safont, Encarnación González Flores, Pilar García-Alfonso, Enrique Aranda, Ana-Maria López Muñoz, Esther Falcó Ferrer, Luís Cirera Nogueras, Nuria Rodríguez-Salas, Jorge Aparicio, Marta Llanos Muñoz, Paola Patricia Pimentel Cáceres, Oscar Alfredo Castillo Trujillo, Rosario Vidal Tocino, Mercedes Salgado Fernández, Antonieta Salud-Salvia, Bartomeu Massuti Sureda, Rocio Garcia-Carbonero, Maria Ángeles Vicente Conesa, Ariadna Lloansí Vila, the PERSEIDA investigators, Manuel Valladares-Ayerbes, Maria José Safont, Encarnación González Flores, Pilar García-Alfonso, Enrique Aranda, Ana-Maria López Muñoz, Esther Falcó Ferrer, Luís Cirera Nogueras, Nuria Rodríguez-Salas, Jorge Aparicio, Marta Llanos Muñoz, Paola Patricia Pimentel Cáceres, Oscar Alfredo Castillo Trujillo, Rosario Vidal Tocino, Mercedes Salgado Fernández, Antonieta Salud-Salvia, Bartomeu Massuti Sureda, Rocio Garcia-Carbonero, Maria Ángeles Vicente Conesa, Ariadna Lloansí Vila

**Affiliations:** 1 Hospital Universitario Virgen del Rocío, Instituto de Biomedicina, 41013 Seville, Spain; 2 Consorcio Hospital General Universitario de Valencia, Universidad de Valencia, Valencia; CIBERONC, Valencia, Spain; 3 Hospital Universitario Virgen de las Nieves, Granada, Spain; 4 Medical Oncology Service, Hospital General Universitario Gregorio Marañón, Instituto de Investigación Sanitaria Gregorio Marañón (IiSGM), Universidad Complutense, Madrid, Spain; 5 Hospital Universitario Reina Sofía, Córdoba, Spain; 6 Hospital Universitario de Burgos, Burgos, Spain; 7 Hospital Son Llàtzer, Palma, Spain; 8 Hospital Universitari Mutua de Terrassa, Terrassa, Spain; 9 Hospital Universitario la Paz, Madrid, Spain; 10 Hospital Universitari i Politècnic La Fe, Valencia, Spain; 11 Hospital Universitario de Canarias, San Cristóbal de La Laguna, Spain; 12 Complejo Hospitalario Area II de Cartagena Hospital Universitario Santa Lucia, Cartagena, Spain; 13 Hospital Universitario Central de Asturias, ISPA, Oviedo, Spain; 14 Complejo Asistencial Universitario de Salamanca, IBSAL, Salamanca, Spain; 15 Complexo Hospitalario Universitario de Ourense, Ourense, Spain; 16 Hospital Universitario Arnau de Vilanova, Lleida, Spain; 17 Hospital Universitario de Alicante, Alicante, Spain; 18 Hospital Universitario 12 de Octubre, Imas12, UCM, Madrid, Spain; 19 Hospital General Universitario José Maria Morales Meseguer, Murcia, Spain; 20 AMGEN S.A., Barcelona, Spain

**Keywords:** Liquid biopsies, ctDNA, mCRC, *RAS*

## Abstract

**Purpose:**

*RAS* (*KRAS*/*NRAS*) mutational status on a tumor biopsy is mandatory to guide the best treatment in metastatic colorectal cancer (mCRC). Determining the *RAS* mutational status by tumor-tissue biopsy is essential in guiding the optimal treatment decision for mCRC. *RAS* mutations are negative predictive factors for the use of EGFR monoclonal antibodies. Cell-free DNA (cfDNA) analysis enables minimally invasive monitoring of tumor evolution.

**Methods/patients:**

PERSEIDA was an observational, prospective study assessing cfDNA *RAS*, *BRAF* and *EGFR* mutations (using Idylla™) in first-line mCRC, *RAS* wild-type (baseline tumor-tissue biopsy) patients (cohort 2). Plasma samples were collected before first-line treatment, after 20 ± 2 weeks, and at disease progression.

**Results:**

117 patients were included (103 received panitumumab + chemotherapy as first-line treatment). At baseline, 7 (6.8%) patients had *RAS* mutations, 4 (3.9%) *BRAF* mutations and no *EGFR* mutations were detected (cfDNA, panitumumab + chemotherapy subpopulation [panitumumab + Ch]). The baseline *RAS* mutational status concordance between tissue and liquid biopsies was 94.0% (93.2%, panitumumab + Ch). At 20 weeks, only one patient in the study (included in the panitumumab + Ch) had an emerging cfDNA *RAS* mutation. No emerging *BRAF* or *EGFR* mutations were reported. At disease progression, 6 patients had emergent mutations not present at baseline (*RAS* conversion rate: 13.3% [6/45]; 15.0% [6/40], panitumumab + Ch).

**Conclusions:**

The concordance rate between liquid and solid biopsies at baseline was very high, as previously reported, while our results suggest a considerable emergence of *RAS* mutations during disease progression. Thus, the dynamics of the genomic landscape in ctDNA may provide relevant information for the management of mCRC patients.

**Supplementary Information:**

The online version contains supplementary material available at 10.1007/s12094-024-03487-4.

## Introduction

Determining the *RAS* (*KRAS/NRAS*) mutational status on a tumor biopsy is mandatory to guide the best treatment recommendation in metastatic colorectal cancer (mCRC). Anti-epidermal growth factor receptor (EGFR) inhibitors in combination with chemotherapy doublet for mCRC treatment is associated with an overall survival that exceeds 36 months in selected populations [1].

Genotyping of *RAS* mutations is routinely done on tumor tissue to predict drug resistance to anti-EGFR in patients with mCRC [2]. The ESMO Clinical Practice Guideline 2022 has included the possibility that in situations where adequate tissue is not available, *RAS* mutation status can be analyzed by plasma-derived cell-free DNA (cfDNA) [1].

Several mechanisms of acquired resistance to anti-EGFR have been described in mCRC. The most common are the emergence of *RAS, BRAF* or *EGFR* mutations [3–5].

The analysis of ctDNA could provide dynamic genetic information about the tumor while the patients are being treated [6–10].

This is an observational and prospective study based on two cohorts of first-line therapy in mCRC. The results of cohort 1 have already been reported [11]. Here, we present the results of cohort 2 by assessing (I) the percentage of patients with *RAS, BRAF* and *EGFR* mutations and (II) the concordance rate for *RAS* mutational status in tumor-tissue and plasma samples (using the Idylla™) at baseline in *RAS* wild-type mCRC patients starting their standard first-line treatment.

## Methods

This was a nationwide, observational, multi-center, prospective study that evaluated the mutational status of *RAS*, *BRAF* and *EGFR* using liquid biopsies (Idylla™ as in vitro diagnostic (IVD) test [Biocartis, Mechelen, Belgium]) [12] in *RAS* wild-type mCRC patients according to baseline tumor-tissue biopsy per local practice (PERSEIDA study, NCT02792478).

The mutational status of *BRAF* at baseline in solid biopsy was also assessed.

The participants were following clinical practice, including the baseline tumor-tissue biopsy and the selection of the first-line treatment. The methodology have been previously published [11].

*RAS* and *BRAF* and *EGFR* mutational status were assessed at baseline, at Week 20 and at disease progression in liquid biopsy (Idylla™). The mutant allele fraction (MAF) threshold for *KRAS* and *BRAF* was 0.4% and for *NRAS* was 1.5%.

Patients fulfilling the following inclusion criteria (as reported for cohort 1 [11] were recruited consecutively and followed-up until disease progression: patients ≥ 18 years, with mCRC measurable by RECIST, who start first-line treatment and with a histologically confirmed diagnosis of mCRC and wild-type *RAS* (according to baseline tumor-tissue biopsy).

Blood samples collection and storage was performed as already described [11]. Additionally, the frozen baseline plasma of 20 patients (subgroup of cohort 2 patients) was shipped to Sysmex Inostics GmbH (Hamburg, Germany) for BEAMing analysis [13] to determine the *RAS* mutation status concordance between the two techniques. The frozen plasma (at disease progression) of 8 of these patients with progressive disease was prospectively selected when sufficient sample was available and shipped to a central laboratory (Instituto Maimónides de Investigación Biomédica, Córdoba, Spain) for analysis using the next-generation sequencing (NGS) technique AVENIO® (cfDNA expanded panel and Illumina NextSeq 550) [14].

The protocol was approved by an independent ethics committee *CEIC Consorcio Hospitalario Provincial de Castellón* (Spain), and all patients gave their written informed consent before enrollment. The study has been performed according to the Declaration of Helsinki.

### Statistical analysis

The primary endpoint was the percentage of patients with *RAS*, *BRAF* and *EGRF* mutations in liquid biopsies at baseline. The percentage of discordant patients and concordance rate (liquid vs solid biopsies results) was calculated for *RAS*, along with its 95% confidence interval (CI). Secondary endpoints included the description of *RAS*, *BRAF* and *EGRF* mutations in liquid biopsies at disease progression and at 20 ± 2 weeks. As an exploratory endpoint, the percentage of concordance rate (liquid vs solid biopsies) and Cohen’s kappa coefficient were also calculated for *BRAS* in the panitumumab subgroup.

The conversion rate for *RAS* at disease progression was assessed as earlier reported [11]. The conversion rates for *BRAF* and *EGFR* were also analyzed*.*

The mutations analyzed in *RAS*, *BRAF* and *EGFR* using Idylla™ are described in Supplementary Table S1.

The association between cfDNA *RAS* and *BRAF* mutational status, respectively, with the overall response rate (ORR) and progression-free survival (PFS) was performed. Moreover, PFS analyses using Kaplan–Meier approach, and a multivariable Cox regression analysis to assess the predictive factors of PFS were also performed as formerly described [11].

Agreement between cfDNA biopsies (Idylla™ vs. BEAMing) for *RAS* at baseline in the subpopulation assessed (*n* = 20) was assessed by Cohen’s kappa coefficient.

Statistical analyses were performed with the SAS statistical software package (SAS Institute, Inc, Cary, NC).

## Results

### Baseline characteristics

Between May 2018 and May 2021, 131 patients were screened in 18 Spanish hospitals (117 patients were included, evaluable population). The most frequent first-line treatment was panitumumab plus chemotherapy (*n* = 103) (panitumumab subpopulation). The main baseline characteristics are shown in Table 1.

**Table 1:** Baseline demographic and clinical characteristics

	Evaluable population^b^ (*n* = 117)	Panitumumab subpopulation^a^ (*n* = 103)
Male, *n* (%)	79 (67.5)	69 (67.0)
Age (years), mean (SD)	65.0 (10.4)	64.8 (10.6)
Ethnic origin: Caucasian, *n* (%)	113 (96.6)	99 (96.1)
BMI (kg/m^2^), mean (SD)	26.4 (4.5)	26.2 (4.3)
Tumor stage at first diagnosis, *n* (%)		
0	1 (0.9)	1 (1.0)
1	1 (0.9)	1 (1.0)
2	10 (8.6)	7 (6.8)
3	17 (14.5)	16 (15.5)
4	87 (74.4)	78 (75.7)
Not available	1 (0.9)	0 (0)
ECOG performance status, *n* (%)		
0	54 (46.2)	47 (45.6)
1	42 (35.9)	36 (35.0)
2	6 (5.1)	6 (5.8)
3	3 (2.6)	3 (2.9)
Not available	12 (10.3)	11 (10.7)
Köhne prognostic score, *n* (%)		
Low risk	46 (39.3)	37 (35.9)
Medium risk	40 (34.2)	37 (35.9)
High risk	12 (10.3)	11 (10.7)
Not available	19 (16.2)	18 (17.5)
Primary tumor location^c^, *n* (%)		
Left colon	89 (76.1)	81 (78.6)
Right colon	27 (23.1)	21 (20.4)
Both (left and right colon)	1 (0.9)	1 (1.0)
Baseline solid biopsy extraction localization, *n* (%)		
Primary	103 (88.0)	90 (87.4)
Metastasis	14 (12.0)	13 (12.6)
Liver	8 (6.8)	8 (7.7)
Peritoneum	5 (4.3)	4 (3.9)
Small intestine	1 (0.9)	1 (1.0)
*BRAF* mutational status (solid biopsy)		
Mutated	5 (4.3)^d^	4 (3.9)
Not mutated	109 (93.2)	96 (93.2)
Not evaluated	3 (2.6)	3 (2.9)
Microsatellite instability/defective MMR (solid biopsy)		
Present	11 (9.40)	11 (10.7)
Absent	38 (32.5)	32 (31.1)
Unknown	1 (0.9)	1 (1.0)
Not evaluated	67 (57.3)	59 (57.3)
Previous surgeries for colorectal cancer, *n* (%)	54 (46.2)	47 (45.6)
Prior treatment for colorectal cancer, *n* (%)	30 (25.6)	27 (26.2)
Radiotherapy	1 (0.9)	1 (1.0)
Chemotherapy	19 (16.2)	17 (16.5)
Radiotherapy and chemotherapy	10 (8.6)	9 (8.7)
No prior treatment	87 (74.4)	76 (73.8)
Affected organs, *n* (%)		
Liver	85 (72.7)	78 (75.7)
Lung	33 (28.2)	32 (31.1)
Lymph nodes	32 (27.4)	29 (28.2)
Peritoneum	29 (24.8)	24 (23.3)
Bone	2 (1.7)	2 (1.9)
Spleen	2 (1.7)	2 (1.9)
Adrenal	1 (0.9)	1 (1.0)
Other	19 (16.2)	16 (15.5)
Target lesions total size (mm), mean (SD)	70.5 (47.7)	73.9 (49.0)
Serum carcinoembryonic antigen (ng/mL), median (Q1, Q3)	15.5 (3.8, 88.5)	16.6 (5.1, 100.2)
Lactate dehydrogenase, ULN, median (Q1, Q3)	247.0 (192.0, 484.0)	254.0 (194.0, 493.0)
Time (months) since solid biopsy extraction to *RAS* wild-type determination, median (Q1, Q3)	0.7 (0.2, 1.5)	0.6 (0.1, 1.3)
Time (months) since *RAS* wild-type determination by a solid biopsy to inclusion, median (Q1, Q3)	0.4 (0.2, 1.0)	0.4 (0.1, 1.1)
Time (months) since histological diagnosis to inclusion, median (Q1, Q3)	1.7 (0.9, 9.0)	1.6 (0.9, 8.0)

*BMI* body mass index, *Q1* 25th percentile, *Q3* 75th percentile, *SD* standard deviation, *ULN* upper limit of normality

^a^Evaluable population treated with chemotherapy plus panitumumab. Among them, 85 patients were treated with panitumumab plus FOLFOX

^b^In the evaluable population, the most commonly initiated chemotherapy regimen was FOLFOX (*n* = 94), followed by FOLFIRI (*n* = 15). Additionally, 6 patients received chemotherapy alone, 5 patients received cetuximab plus chemotherapy and 3 patients received bevacizumab plus chemotherapy

^c^As left colon localization, the following terms were included: left colon, sigmoid colon and/or rectum

^d^V600D (c.1799_1800TG>AC): *n* = 1; V600E (c.1799T>A;c.1799_1800TG>AC): *n* = 3; undefined: *n* = 1

### Liquid biopsy (Idylla™) results

In the evaluable population, 7 (6.0%) patients presented *RAS* mutations in cfDNA at baseline. Additionally, 6 (5.1%) patients had *BRAF* mutations, while *EGFR* mutations were not detected. At baseline, the percentage of *RAS* mutational status concordance between tissue and liquid biopsies was 94.0% (95% CI 88.1–97.6) with comparable results in the panitumumab subpopulation (Table 2).

**Table 2:** Liquid biopsy results (Idylla™)

Baseline	Evaluable population	Panitumumab subpopulation
	*n* = 117	*n* = 103
*RAS* mutational status, *n* (%)		
Wild-type	110 (94.0)	96 (93.2)
Mutant	7 (6.0)	7 (6.8)
*KRAS* mutant	5 (4.3)	5 (4.9)
*NRAS* mutant	2 (1.7)	2 (1.9)
*RAS* mutant detection rate, % (95% CI)^a^	6.0 (2.4–11.9)	6.8 (2.8–13.5)
Negative percent agreement (*RAS*), % (95% CI)^b^	94.0 (88.1–97.6)	93.2 (86.5–97.2)
*BRAF* mutational status, *n* (%)		
Wild-type	106 (90.6)	94 (91.3)
Mutant	6 (5.1)^c^	4 (3.9)
Missing	5 (4.3)	5 (4.9)
Negative percent agreement (*BRAF*), % (95% CI)^b^	–	98.9 (94.0–100.0)
*EGFR* mutational status, n (%)		
Wild-type	74 (63.3)	65 (63.1)
Mutant	0 (0.0)	0 (0.0)
Missing	43 (36.8)	38 (36.9)

At 20 weeks	*n* = 83	*n* = 74
*RAS* mutational status, *n* (%)		
Wild-type	81 (97.6)	72 (97.3)
Mutant	2 (2.4)	2 (2.7)
*KRAS* mutant	2 (2.4)	2 (2.7)
*NRAS* mutant	0 (0.0)	0 (0.0)
*BRAF* mutational status, *n* (%)		
Wild-type	77 (92.8)	69 (93.2)
Mutant	3 (3.6)^d^	2 (2.7)
Missing	3 (3.6)	3 (4.1)
*EGFR* mutational status, *n* (%)		
Wild-type	39 (47.0)	33 (44.6)
Mutant	0 (0.0)	0 (0.0)
Missing	44 (53.0)	41 (55.4)

At disease progression	*n* = 50	*n* = 45
*RAS* mutational status, *n* (%)		
Wild-type	42 (84.0)	37 (82.2)
Mutant	7 (14.0)	7 (15.6)
*KRAS* mutant	5 (10.0)	5 (11.1)
*NRAS* mutant	2 (4.0)	2 (4.4)
*BRAF* mutational status, *n* (%)		
Wild-type	38 (76.0)	35 (77.8)
Mutant	3 (6.0)^e^	1 (2.2)
Missing	9 (18.0)	9 (20.0)
*EGFR* mutational status, *n* (%)		
Wild-type	10 (20.0)	8 (17.8)
Mutant	0 (0.0)	0 (0.0)
Missing	40 (80.0)	37 (82.2)

*CI* confidence interval using the Clopper–Pearson exact method

^a^Percentage of discordant patients

^b^Percentage of concordant patients in patients *RAS or BRAF* wild-type (as appropriate) according to solid biopsy

^c^All 6 patients presented V600D (c.1799_1800delinsAC) OR V600E (c.1799T>A;c.1799_1800delinsAA) mutations

^d^2 patients presented V600D (c.1799_1800delinsAC) OR V600E (c.1799T>A;c.1799_1800delinsAA) mutations and 1 patient presented V600K (c.1798_1799delinsAA) OR V600R (c.1798_1799delinsAG) mutations

^e^All 3 patients presented V600D (c.1799_1800delinsAC) OR V600E (c.1799T>A;c.1799_1800delinsAA) mutations

At baseline, the percentage of *BRAF* mutational status concordance between tissue and liquid biopsies was 98.9% (95% CI 94.0–100.0) (Table 2). The Cohen’s Kappa coefficient was 0.739 (95% CI 0.392–1.000), indicative of a substantial agreement.

A logistic regression analysis did not find any variable (liver metastasis, tumor burden, number of affected organs at study entry, primary tumor surgery and serum carcinoembryonic antigen [CEA] levels) associated with discordant cases at baseline (data not shown).

Additionally, the overall percent *RAS* agreement between the two tests (Idylla™ vs. BEAMing) was 90.0% (Cohen’s kappa = 0.444, indicative of a moderate agreement; *n* = 18) considering a mutant allele fraction ≥ 0.02% as a threshold.

At 20 weeks, 83 patients had blood sample available, 2 of them (2.4%) presented cfDNA *RAS* mutations in the evaluable population (both received panitumumab plus chemotherapy) and 3 (3.6%) presented *BRAF* mutations (2 received panitumumab plus chemotherapy and 1 received bevacizumab plus chemotherapy). *EGFR* mutations were not detected.

At disease progression, 7/50 (14.0%) patients with blood sample available presented cfDNA *RAS* mutations in the evaluable population (all 7 received panitumumab plus chemotherapy) and 3 (6.0%) had *BRAF* mutations (1 received panitumumab plus chemotherapy). *EGFR* mutations were not detected (Table 2).

Regarding the emerging mutations, 6 patients in the panitumumab subpopulation presented *RAS* mutations in cfDNA at disease progression that were not present at baseline (Table 3). Accordingly, the *RAS* conversion rate at disease progression was 13.3% (6/45 patients with baseline *RAS* wild-type status, both by tumor and cfDNA analysis) and blood sample available at disease progression) in the evaluable population and 15.0% (6/40 patients) in the panitumumab subpopulation. At 20 weeks, 1 patient had emergent *RAS* mutations (received panitumumab plus chemotherapy) (Table 3). No patients had emergent *BRAF* or *EGFR* mutations.

**Table 3:** Characteristics of patients with emergent *RAS* mutations at disease progression or at week 20 (liquid biopsy, Idylla™)

Patient	*RAS* mutant			Gene mutated: codon-exon-nucleotide position	Primary tumor location	Site of metastasis	Days^a^	Best overall response^b^	PFS (months)
	Baseline	Week 20	Disease progression						
1	Wild-type	Wild-type	Mutant	*NRAS:* codon 12 (exon 2)-G12A (c35G>C) *NRAS:* codon 12 (exon 2)-G12V (c35G>T)	Right colon	Liver, lung, peritoneum	7	PD	4.8
2	Wild-type	Wild-type	Mutant	*KRAS:* codon 61 (exon 3)-Q61H (c183A>C; c183A>T)	Left colon	Liver	16	PR	13.0
3	Wild-type	Wild-type	Mutant	*KRAS:* codon 12 (exon 2)-G12D (c35G>A)	Left colon	Liver	13	PR	9.3
4	Wild-type	Wild-type	Mutant	*NRAS:* codon 61 (exon 3)-Q61K (c181C>A) *NRAS:* codon 61 (exon 3)-Q61R (c182A>G)	Left colon	Peritoneum, others	51	CR	32.6
5	Wild-type	Wild-type	Mutant	*KRAS:* codon 61 (exon 3)-Q61H (c183A>C; c183A>T)	Left colon	Peritoneum	351	SD	5.3
6	Wild-type	Wild-type	Mutant	*KRAS:* codon 61 (exon 3)-Q61H (c183A>C; c183A>T)	Left colon	Liver, lymph nodes, others	8	PR	19.2
7	Wild-type	Mutant	No PD	*KRAS:* codon 12 (exon 2)-G12D (c35G>A)	Left colon	Liver	7	CR	14.7

*CR* complete response, *PFS* progression-free survival, *PR* partial response, *SD* stable disease

^a^Days between tissue and blood sample collection

^b^Not confirmed response

The characteristics of patients with mutations at any time (per liquid biopsy) are shown in Table 4 (*RAS* mutations) and in Supplementary Table S2 (*BRAF* mutations). Among the 6 patients with emerging *RAS* mutations at disease progression, 4 achieved complete or partial response as the best overall response, while 1 achieved stable disease and 1 progressive disease. In these patients, the most frequent metastatic site was liver (*n* = 4).

**Table 4:** Characteristics of patients with *RAS* mutations at any time as per liquid biopsy (Idylla™)

Patient	*RAS* mutant			Primary tumor location	Site of metastasis	Days^a^	First-line treatment	Best overall response^b^	PFS (months)
	Baseline	Week 20	Disease progression						
1	Mutant	Wild-type	Wild-type	Left colon^c^	Liver, lung, lymph nodes	188	FOLFIRI + panitumumab	PR	8.0
2	Wild-type	Wild-type	Mutant	Right colon	Liver, lung, peritoneum	7	FOLFOX + panitumumab	PD	4.8
3	Mutant	Not Available	Wild-type	Right colon	Liver, peritoneum	34	FOLFOX + panitumumab	PD	2.5
4	Mutant	Wild-type	No PD	Left colon^c^	Liver	11	FOLFOX + panitumumab	CR	27.5
5	Wild-type	Wild-type	Mutant	Left colon^c^	Liver	16	FOLFOX + panitumumab	PR	13.0
6	Mutant	Not Available	PD, but sample not available	Left colon^c^	Liver	652	FOLFIRI + panitumumab	SD	9.7
7	Mutant	Wild-type	PD, but sample not available	Right colon	Liver, lung	40	FOLFOX + panitumumab	PR	13.7
8	Wild-type	Wild-type	Mutant	Left colon^c^	Liver	13	FOLFOX + panitumumab	PR	9.3
9	Mutant	Mutant	Mutant	Right colon	Liver, peritoneum	201	FOLFOX + panitumumab	PR	6.2
10	Wild-type	Wild-type	Mutant	Left colon^c^	Peritoneum, other	51	FOLFOX + panitumumab	CR	32.6
11	Mutant	Wild-type	Wild-type	Right colon	Liver, peritoneum, other	546	FOLFIRI + panitumumab	PR	10.9
12	Wild-type	Wild-type	Mutant	Left colon^c^	Peritoneum	351	FOLFIRI + panitumumab	SD	5.3
13	Wild-type	Mutant	No PD	Left colon^c^	Liver	7	FOLFOX + panitumumab	CR	14.7
14	Wild-type	Wild-type	Mutant	Left colon^c^	Liver, lymph nodes, other	8	FOLFOX + panitumumab	PR	19.2

*CR* complete response, *PD* progressive disease, *PFS* progression-free survival, *PR* partial response, *SD* stable disease

^a^Days between tissue and sample collection

^b^Not confirmed response

^c^As left colon localization, the following terms were included: left colon, sigmoid colon and/or rectum

Three patients with *RAS* mutations in liquid biopsies at baseline were *RAS* wild-type at disease progression (Table 4, patients #1, #3 and #11).

### Association with patient outcomes (panitumumab subpopulation)

The ORR (not confirmed) was 80.6% (95% CI 71.4–87.9) (*n* = 98 data available), being 85.7% (66/77) and 63.6% (14/22) in patients with left and right-sided primary tumor, respectively. In this subpopulation of panitumumab plus FOLFOX, the ORR (not confirmed) was 83.8% (95% CI 73.8–91.1%).

The ORR according to cfDNA *RAS*, *BRAF* and *RAS*/*BRAF* mutational status at baseline and by primary tumor location is displayed in Table 5. Among patients receiving panitumumab, the ORR did not show statistical differences regardless of the cfDNA *RAS* and *BRAF* mutational status at baseline. However, the odds ratio was greater than 1, indicating a trend towards a higher probability of response (ORR) in cfDNA wild-type patients compared to those with *RAS* or *BRAF* mutations. This trend was higher in left-sided vs right-sided tumors (*RAS* and *RAS/BRAF* mutants).

**Table 5:** ORR by *RAS*, *BRAF*, and *RAS/BRAF* mutational status in liquid biopsy at baseline (panitumumab subpopulation, total and by primary tumor location)

*RAS*	Wild-type	Mutant	Total
	*n* = 91	*n* = 7	*n* = 98
ORR, % (95% CI)	81.3% (71.8–88.7)	71.4% (29.0–96.3)	80.6% (71.4–87.9)
Odds ratio (95% CI*)			1.74 (0.31–9.75)
Left-sided tumors	*n* = 74	*n* = 3	*n* = 77
ORR, % (95% CI)	86.5% (76.6–93.3)	66.7% (9.4–99.2)	85.7% (75.9–92.7)
Odds ratio (95% CI*)			3.20 (0.26–38.64)
Right-sided tumors	*n* = 18	*n* = 4	*n* = 22
ORR, % (95% CI)	61.1% (35.8–82.7)	75.0% (19.4–99.4)	63.6% (40.7–82.8)
Odds ratio (95% CI*)			0.52 (0.05–6.09)

*BRAF*	*n* = 89	*n* = 4	*n* = 93
ORR, % (95% CI)	82.0% (72.5–89.4)	75.0% (19.4–99.4)	81.7% (72.4–89.0)
Odds ratio (95% CI*)			1.52 (0.15–15.58)
Left-sided tumors	*n* = 74	*n* = 1	*n* = 75
ORR, % (95% CI)	87.8% (78.2–94.3)	0% (0–97.5)	86.7% (76.8–93.4)
Odds ratio (95% CI*)			–
Right-sided tumors	*n* = 16	*n* = 3	*n* = 19
ORR, % (95% CI)	56.3% (29.9–80.3)	100% (29.2–100)	63.2% (38.4–83.7)
Odds ratio (95% CI*)			–

*RAS*/*BRAF*	*n* = 87^a^	*n* = 11^b^	*n* = 98
ORR, % (95% CI)	81.6% (71.9–89.1)	72.7% (39.0–94.0)	80.6% (71.4–87.9)
Odds ratio (95% CI*)			1.66 (0.40–6.98)
Left-sided tumors	*n* = 73^a^	*n* = 4^b^	*n* = 77
ORR, % (95% CI)	87.7% (77.9–94.2)	50.0% (6.8–93.2)	85.7% (75.9–92.7)
Odds ratio (95% CI*)			7.11 (0.89–56.95)
Right-sided tumors	*n* = 15^a^	*n* = 7^b^	*n* = 22
ORR, % (95% CI)	53.3% (26.6–78.7)	85.7% (42.1–99.6)	63.6% (40.7–82.8)
Odds ratio (95% CI*)			0.19 (0.02–1.99)

Not confirmed response. One patient presented with both left and right-sided colon tumor as primary location

*CI* confidence interval, *ORR* overall response rate

*No statistical differences when 95% CI of odds ratio contains 1

^a^Patients with both *RAS* and *BRAF* wild-type as per liquid biopsy at baseline

^b^Patients with either *RAS* or *BRAF* mutant as per liquid biopsy at baseline

Regarding PFS, the median (95% CI) time was 12.5 (9.9–13.8) months. There were no statistically significant differences in median PFS according to cfDNA *RAS*, *BRAF* or *RAS*/*BRAF* mutational status at baseline or at any time (Table S3). There were also no differences in median PFS between patients who were always *RAS* wild-type (as per cfDNA) and patients with *RAS* mutations at any time. Similar results were observed by *RAS* status at baseline or at any time in the subgroup of patients with left-sided tumors (Table S4). Additionally, related to the tumor laterality, no differences in median PFS (between patients either cfDNA *RAS/BRAF* mutations at any time or NGS mutations or baseline microsatellite instability/defective MMR vs patients without these alterations) were found, either in the total population or in the panitumumab subgroup of patients with left-sided tumors (Supplementary Fig S1 and Fig. S2, respectively). The Cox regression analysis did not find any predictive factor of PFS (data not shown). In this subpopulation of panitumumab plus FOLFOX, the median PFS (95% CI) time was 13.3 (10.9–15.0) months.

The multivariable model to predict tumor burden (defined as the sum of the longest tumor diameters [mm]) (which included as predictable factors: liver metastasis, *RAS* and *BRAF* mutant/wild-type status at baseline) showed that the presence of *RAS* or *BRAF* mutations at baseline was not associated with tumor burden but liver metastasis vs not having liver metastasis was significantly associated with tumor burden (difference of 41.5 mm between them, 95% CI 19.2–63.7; *p* = 0.0004) (Supplementary Table S5).

### NGS results

Subclonal genomic variants potentially associated with anti-EGFR resistance were found in 6/8 patients tested (75%). More details are described in Supplementary Table S6.

## Discussion

The PERSEIDA study was a prospective, multicentric and observational study. This prospective approach design has allowed us to get a homogeneous sample with 92% of patients receiving anti-EGFR plus chemotherapy as first-line treatment. Among those, 97% received panitumumab plus chemotherapy, being an informative population for this treatment.

A high concordance for *RAS* status between tissue and plasma samples (using Idylla™) was observed, being comparable to that previously published in cohort 1 using the BEAMing technique [11]. Moreover, a high percent agreement between both cfDNA tests (Idylla™ and BEAMing techniques was reported (90%, considered as moderate concordance according to Cohen’s kappa). It should be highlighted that Idylla™ is a technique widely used in many centers for the determination of the *RAS* mutational status in routine practice, so these results are highly informative for clinical practice in patients with mCRC.

Emergent *RAS* mutations were mostly observed at disease progression (in 6 patients). At 20 weeks, only 1 patient presented emergent *RAS* mutations. Emergent *RAS* mutations were located in exon 2 and 3 of both *KRAS* and *NRAS,* with a similar frequency between them. Nevertheless, the prevalence of *RAS* mutations in ctDNA was reported to be higher for *KRAS* exon 2 compared to the other locations (43% vs 3–4%) [15]. Of note, a trend of emerging Q61 mutations in *KRAS* which were described as infrequent [16]. As the number of patients with emergent mutations in this study is low, comparison with other studies is difficult.

The highest proportion of patients with *RAS* mutations at disease progression could explain only in part the appearance of acquired resistance to first-line treatment. Some studies reported a similar rate of emerging *RAS* mutations during first-line treatment with anti-EGFR plus chemotherapy [4, 17]. Diaz *et al.* [7] suggested that resistance mutations were highly likely to be present in a clonal subpopulation prior to the initiation of therapy and the time to recurrence was simply the interval required for the subclone to re-populate the lesion. Aligned to this, Takayama *et al.* [6] also proposed that latent cells from tumors, with undetected *KRAS* mutations may undergo clonal expansion during the treatment. Other authors reported that *RAS* mutations can be attributed not only to the selection of pre-existing *RAS* clones but also to the mutant clone as the result of de novo acquisition of a *RAS* mutation [10]. By contrast Parseghian *et al.* [17] suggested that rather than an outgrowth of preexisting clones, a transcriptional mechanism of acquired resistance appears to predominate in patients treated with a combination of an anti-EGFR and cytotoxic chemotherapy at first line. Therefore, we hypothesize in line with prior research that the identification of *RAS* mutations in ctDNA might have higher clinical relevance in later lines of therapy than in baseline first-line setting, acknowledging the high concordance observed with tissue biopsy and low appearance of emergent *RAS* mutations during first-line treatment.

By contrast to the emergent *RAS* mutations, there were three patients with *RAS* mutations at baseline (according to liquid biopsy) that were *RAS* wild-type at progression. The explanation is unknown, however, the disappearance of *RAS* mutant clones in plasma has been described before, supporting a negative selection of *RAS* mutations during the clonal evolution of mCRC. Nevertheless, the extent of conversion to *RAS* wild-type disease at the time of progression has not been clarified yet [18]. Another explanation could be the sensitivity of the liquid biopsy as ctDNA often represents only a small fraction of total cfDNA [19–21]. Other factors as assay type, technical and biological background can also affect *RAS* mutational detection.

The NGS results found subclonal genomic variants potentially associated with anti-*EGFR* resistance in 6 out of 8 patients (75%) at disease progression. The NGS analysis suggests that resistance is driven by a complex process of genomics alterations, and not just by *RAS* mutations. The ORR results showed no statistically significant differences according to baseline cfDNA *RAS* mutational status. Despite these, there was a trend towards a higher probability of response in wild-type compared to *RAS* and/or *BRAF* mutational status. This trend was higher in the left-sided tumors compared to right-sided one (*RAS* and *RAS*/*BRAF* mutants). These better results in left-sided tumors were previously reported, suggesting the importance of the primary tumor location [22–24]. Comparable results were reported when considering the *RAS* and *BRAF* mutational status at any time. It should be noted that the presence of baseline mutations in cfDNA had no impact on ORR to chemotherapy plus panitumumab. These results make us consider what the threshold of sensitivity would be, for example what fraction of *RAS* allelic variants in cfDNA, would be clinically relevant to select or not a treatment that includes anti-EGFRs.

Related to the PFS, there were also no significant differences in PFS according to baseline cfDNA *RAS* mutational status or between patients that were always *RAS* wild-type (as per cfDNA) and patients with *RAS* mutant any time point, although a numerically higher trend was observed in *RAS* wild-type patients. The comparison of these results with cohort 1 of the same study that used BEAMing analysis showed that, despite no significant association between ORR or PFS and cfDNA *RAS* mutational status was observed, patients with left-sided tumor presented a median PFS significantly longer among cfDNA *RAS* wild-type patients than those presenting cfDNA *RAS* mutations at any time [11]. The discrepancies between the two cohorts could be done to the different sensitivity between Idylla™ and BEAMing analysis [25]. Even when we selected the patient population most theoretically susceptible of being benefited from anti-EGFR treatment (patients presenting either cfDNA *RAS/BRAF* mutations at any time or NGS mutations or baseline microsatellite instability/defective MMR), we did not find statistically significant differences in PFS, probably due to a low number of patients available and the low Idylla™ sensitivity. In addition, patients who gained new *RAS/BRAF* mutations showed a similar prognosis as those who maintained *RAS/BRAF* mutations, and shorter PFS and OS than those who remained *RAS/BRAF* wild-type [26].

In our multicentric hospital setting, FOLFOX plus panitumumab was the most common first-option treatment for patients with *RAS* wild-type mCRC, with an ORR of 84% and a median PFS time of 13 months. These data are according to previous published results in this population [27, 28].

This study has some limitations. The study presents a large series of patients with native *RAS* tumors who were treated with chemotherapy and anti-EGFR, specifically panitumumab, as a first-line. However, due to the low frequency of *RAS* mutations in cfDNA, it is challenging to obtain clinically and statistically significant differences. Moreover, this study was not initially designed to determine the association between *RAS* status and outcomes and the predictive factors study that were only explorative endpoints.

Some of the strengths are the homogeneity of the prospective studied population, with all the patients being tumor *RAS* wild-type at baseline, starting their first-line treatment (mostly panitumumab plus chemotherapy). This study compared two highly sensitive techniques for cfDNA (Idylla™ and BEAMing techniques). Finally, it assessed not only *RAS* mutational status but also *BRAF* and *EGFR* mutational status over time.

In conclusion, the concordance rate between liquid and solid biopsies at baseline was very high, as previously reported. The emergence of *RAS* mutations during disease progression is noteworthy but may only partially explain acquired resistance to anti-EGFRs, as more complex mechanisms are involved. Therefore, the dynamics of the genomic landscape in ctDNA may provide relevant information for the management of mCRC patients both in first-line and later lines.

## Supplementary Information

Below is the link to the electronic supplementary material.

Supplementary file 1—Fig. S1. Kaplan–Meier curve for time to PFS by mutational status* (panitumumab subpopulation). *Mutational status (any alteration) at any time; PFS: progression free survival (TIF 89 KB)

Supplementary file 2—Fig. S2. Kaplan–Meier curve for time to PFS by mutational status* (patients with left colon localization, panitumumab subpopulation). *Mutational status (any alteration) at any time; PFS: progression free survival (TIF 91 KB)

Supplementary file 3 (DOCX 20 KB)

Supplementary file 4 (DOCX 20 KB)

Supplementary file 5 (DOCX 19 KB)

Supplementary file 6 (DOCX 19 KB)

Supplementary file 7 (DOCX 18 KB)

Supplementary file 8 (DOCX 20 KB)

## Data Availability

The datasets generated during and/or analysed during the current study are available from the corresponding author upon reasonable request.
