# Supplementary figures and images for: Sequential RAS mutations evaluation in cell-free DNA of patients with tissue RAS wild-type metastatic colorectal cancer: the PERSEIDA (Cohort 2) study
Source: Clin Transl Oncol. 2024 Apr 20;26(10):2640–51. doi: 10.1007/s12094-024-03487-4 (PMC11410833; doi:10.1007/s12094-024-03487-4)

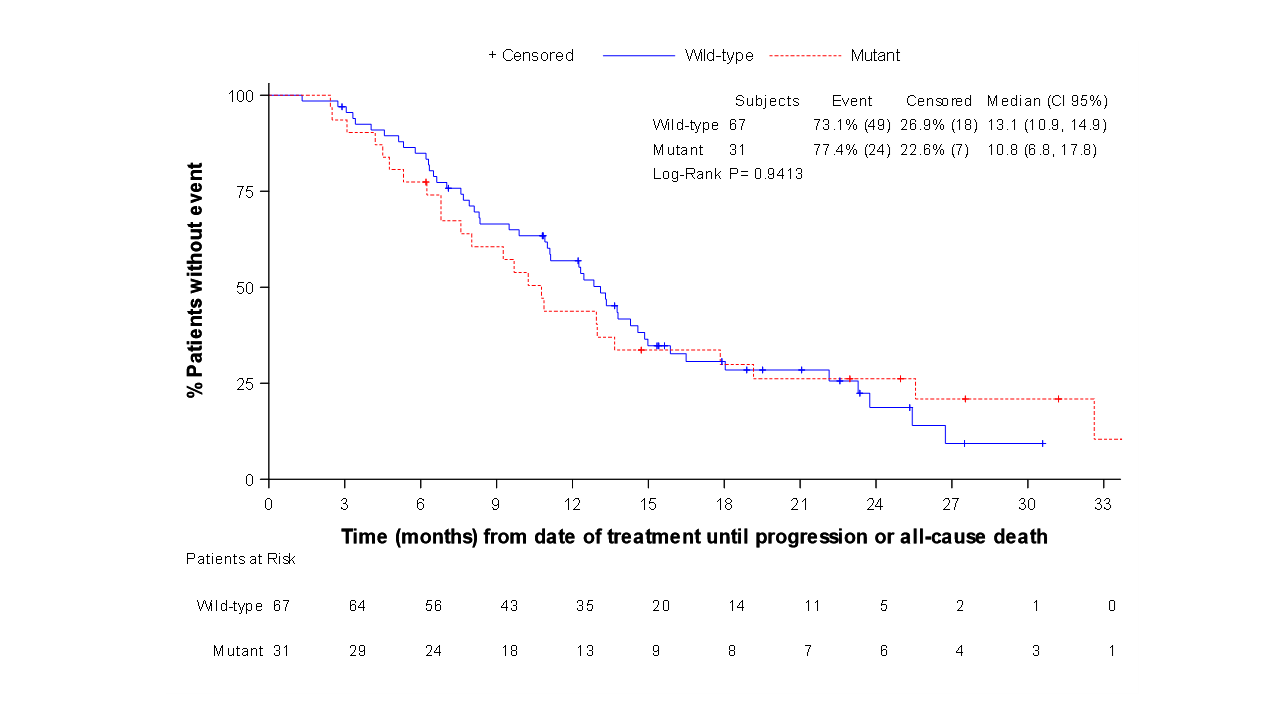

Supplement: Supplementary file 1 — Fig. S1. Kaplan–Meier curve for time to PFS by mutational status* (panitumumab subpopulation). *Mutational status (any alteration) at any time; PFS: progression free survival (TIF 89 KB) [file 12094_2024_3487_MOESM1_ESM.tif]

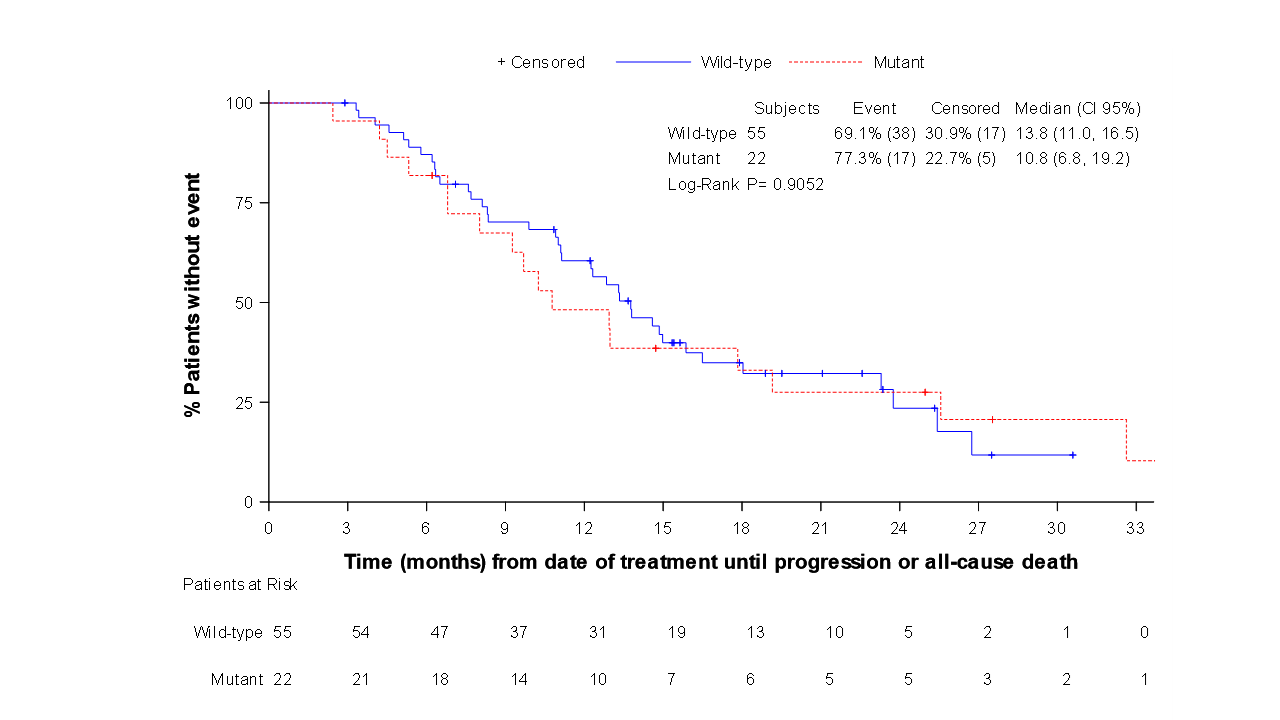

Supplement: Supplementary file 2 — Fig. S2. Kaplan–Meier curve for time to PFS by mutational status* (patients with left colon localization, panitumumab subpopulation). *Mutational status (any alteration) at any time; PFS: progression free survival (TIF 91 KB) [file 12094_2024_3487_MOESM2_ESM.tif]
