# Supplementary material for: Sequential RAS mutations evaluation in cell-free DNA of patients with tissue RAS wild-type metastatic colorectal cancer: the PERSEIDA (Cohort 2) study
Source: Clin Transl Oncol. 2024 Apr 20;26(10):2640–51. doi: 10.1007/s12094-024-03487-4 (PMC11410833; doi:10.1007/s12094-024-03487-4)
Supplement: Supplementary file 3 — (DOCX 20 KB) [file 12094_2024_3487_MOESM3_ESM.docx]

## Sequential *RAS* mutations evaluation in cell-free DNA of patients with tissue *RAS* wild-type metastatic colorectal cancer: The PERSEIDA (Cohort 2) Study

Clinical and Translational Oncology

## Manuel Valladares-Ayerbes, Maria José Safont, Encarnación González Flores, Pilar García-Alfonso, Enrique Aranda, Ana-Maria López Muñoz, Esther Falcó Ferrer, Luís Cirera Nogueras, Nuria Rodríguez-Salas, Jorge Aparicio, Marta Llanos Muñoz, Paola Patricia Pimentel Cáceres, Oscar Alfredo Castillo Trujillo, Rosario Vidal Tocino, Mercedes Salgado Fernández, Antonieta Salud-Salvia, Bartomeu Massuti Sureda, Rocio Garcia-Carbonero, Maria Ángeles Vicente Conesa, Ariadna Lloansí Vila, on behalf of the PERSEIDA investigators

Manuel Valladares Ayerbes

Hospital Universitario Virgen del Rocío, Instituto de Biomedicina, Sevilla, Spain

Email: [mvalaye@icloud.com](mailto:mvalaye@icloud.com)

## Table S1. Mutations analysed (Idylla^TM^)

| *KRAS* |  |
| --- | --- |
| Exon 2 | Codon 12 (mutations: c.34G>A, c.34G>C, c.34G>A, c.35G>C, c.35G>A, c.35G>T) and codon 13 (c.38G>A) |
| Exon 3 | Codon 59 (c.176C>A, c.176C>G, c.175G>A), and 61 (c c.181C>A, c.180_181delinsAA, c.182A>T, c.182A>G, c.183A>C; c.183A>T); exon 4, codons 117 (c.351A>C, c.351A>T) and 146 (c.436G>C, c.436G>A, c.437C>T) |
| Exon 4 | Codon 117 (c.351A>C, c.351A>T) and codon 146 (c.436G>C, c.436G>A, c.437C>T) |
| ***NRAS*** |  |
| exon 2 | Codon 12 (c.34G>T, c.34G>A, c.35G>A, c.35G>C, c.35G>T) and codon 13 (c.38G>A, c.38G>T, c.37G>C) |
| exon 3 | Codon 59 (c.175G>A) and codon 61 (c.181C>A, c.182A>G, c.182A>T, c.183A>T, c.183A>C) |
| exon 4 | Codon 117 (c.351A>C, c.351G>T) and codon 146 (c.436G>A, c.437C>T) |
| ***BRAF*** |  |
|  | Codon 600 (c.1799T>A; c.1799_1800delinsAA, c.1799_1800delinsAC, c.1798_1799delinsAA, c.1798_1799delinsAG) |
| ***EGFR*** |  |
|  | Codon 492 (c.35G>A, c.1474A>C) |
