# Supplementary material for: Sequential RAS mutations evaluation in cell-free DNA of patients with tissue RAS wild-type metastatic colorectal cancer: the PERSEIDA (Cohort 2) study
Source: Clin Transl Oncol. 2024 Apr 20;26(10):2640–51. doi: 10.1007/s12094-024-03487-4 (PMC11410833; doi:10.1007/s12094-024-03487-4)
Supplement: Supplementary file 4 — (DOCX 20 KB) [file 12094_2024_3487_MOESM4_ESM.docx]

## Sequential *RAS* mutations evaluation in cell-free DNA of patients with tissue *RAS* wild-type metastatic colorectal cancer: The PERSEIDA (Cohort 2) Study

Clinical and Translational Oncology

## Manuel Valladares-Ayerbes, Maria José Safont, Encarnación González Flores, Pilar García-Alfonso, Enrique Aranda, Ana-Maria López Muñoz, Esther Falcó Ferrer, Luís Cirera Nogueras, Nuria Rodríguez-Salas, Jorge Aparicio, Marta Llanos Muñoz, Paola Patricia Pimentel Cáceres, Oscar Alfredo Castillo Trujillo, Rosario Vidal Tocino, Mercedes Salgado Fernández, Antonieta Salud-Salvia, Bartomeu Massuti Sureda, Rocio Garcia-Carbonero, Maria Ángeles Vicente Conesa, Ariadna Lloansí Vila, on behalf of the PERSEIDA investigators

Manuel Valladares Ayerbes

Hospital Universitario Virgen del Rocío, Instituto de Biomedicina, Sevilla, Spain

Email: [mvalaye@icloud.com](mailto:mvalaye@icloud.com)

## Table S2. Characteristics of patients with *BRAF* mutations at any time as per liquid biopsy (Idylla^TM^)

| **Patient** | ***BRAF* mutant** | | | | **Primary tumor location** | | **Site of metastasis** | **Days^a^** | **First-line treatment** | **Best overall response^b^** | **PFS (months)** |
| --- | --- | --- | --- | --- | --- | --- | --- | --- | --- | --- | --- |
|  | **Baseline** | **Week 20** | **Disease progression** |  | |  | |  |  |  |  |
| 1 | Mutant | Wild-type | no PD | Right colon | | Liver, Lymph nodes | | 3 | CAPOX + Panitumumab | PR | 31.2 |
| 2 | Mutant | Not Available | PD, but sample not available | Left colon | | Liver | | 35 | FOLFOX + Panitumumab | PD | 2.4 |
| 3 | Mutant | Not Available | no PD | Right colon | | Liver, Lung | | 12 | FOLFOX + Panitumumab | PR | 3.1 |
| 4 | Wild-type | Mutant | PD, but sample not available | Right colon | | Liver, Lung | | 40 | FOLFOX + Panitumumab | PR | 13.7 |
| 5 | Mutant | Mutant | Mutant | Right colon | | Liver, Other | | 36 | FOLFOX + Panitumumab | PR | 7.6 |
| 6 | Mutant | Mutant | Mutant | Right colon | | Peritoneum | | 9 | FOLFOXIRI + Bevacizumab | SD | 8.0 |
| 7 | Mutant | Wild-type | Mutant | Right colon | | Liver, Peritoneum | | 67 | FOLFOX | SD | 16.6 |

a. Days between tissue and sample collection; b. Not confirmed response

Abbreviations: CR; complete response; NE, non-evaluable; PD, progressive disease; PFS, progression free survival; PR, partial response; SD, stable disease.
