# Supplementary material for: Sequential RAS mutations evaluation in cell-free DNA of patients with tissue RAS wild-type metastatic colorectal cancer: the PERSEIDA (Cohort 2) study
Source: Clin Transl Oncol. 2024 Apr 20;26(10):2640–51. doi: 10.1007/s12094-024-03487-4 (PMC11410833; doi:10.1007/s12094-024-03487-4)
Supplement: Supplementary file 5 — (DOCX 19 KB) [file 12094_2024_3487_MOESM5_ESM.docx]

## Sequential *RAS* mutations evaluation in cell-free DNA of patients with tissue *RAS* wild-type metastatic colorectal cancer: The PERSEIDA (Cohort 2) Study

Clinical and Translational Oncology

## Manuel Valladares-Ayerbes, Maria José Safont, Encarnación González Flores, Pilar García-Alfonso, Enrique Aranda, Ana-Maria López Muñoz, Esther Falcó Ferrer, Luís Cirera Nogueras, Nuria Rodríguez-Salas, Jorge Aparicio, Marta Llanos Muñoz, Paola Patricia Pimentel Cáceres, Oscar Alfredo Castillo Trujillo, Rosario Vidal Tocino, Mercedes Salgado Fernández, Antonieta Salud-Salvia, Bartomeu Massuti Sureda, Rocio Garcia-Carbonero, Maria Ángeles Vicente Conesa, Ariadna Lloansí Vila, on behalf of the PERSEIDA investigators

Manuel Valladares Ayerbes

Hospital Universitario Virgen del Rocío, Instituto de Biomedicina, Sevilla, Spain

Email: [mvalaye@icloud.com](mailto:mvalaye@icloud.com)

## Table S3. PFS according to *RAS*, *BRAF*, and *RAS/BRAF* mutational status in liquid biopsy at baseline and at any time (panitumumab subpopulation)

|  | **Wild-type** | **Mutant** | **Total** |
| --- | --- | --- | --- |
| ***At baseline*** |  |  |  |
| ***RAS*** | **n = 91** | **n = 7** | **n = 98** |
| PFS, % (95% CI) | 12.9 (10.3-14.6) | 9.7 (2.5-13.7) | 12.5 (9.9-13.8) |
| p-value |  |  | 0.429 |
| ***BRAF*** | **n = 89** | **n = 4** | **n = 93** |
| PFS, % (95% CI) | 12.9 (10.8 - 14.6) | 5.3 (2.4-) | 12.9 (10.3-14.3) |
| p-value |  |  | 0.650 |
| ***RAS* / *BRAF*** | **n = 87** | **n = 11** | **n = 98** |
| PFS, % (95% CI) | 12.9 (10.8-14.6] | 8.0 (2.5-13.7) | 12.5 (9.9-13.8) |
| p-value |  |  | 0.342 |
| ***At any time*** | **Wild-type** (always) | **Mutant** (at any time) | **Total** |
| ***RAS*** | **n = 84** | **n = 14** | **n = 98** |
| PFS, % (95% CI) | 12.85 (10.25 - 14.59) | 10.28 (5.32 - 19.15) | 12.45 (9.89 - 13.80) |
| p-value |  |  | 0.626 |
| ***BRAF*** | **n = 91** | **n = 5** | **n = 96** |
| PFS, % (95% CI) | 12.9 (10.8-14.6) | 7.6 (2.4-) | 12.85 (10.3-13.8) |
| p-value |  |  | 0.602 |
| ***RAS* / *BRAF*** | **n = 80** | **n = 18** | **n = 98** |
| PFS, % (95% CI) | 12.9 (10.9-14.6) | 9.5 (5.3-13.7) | 12.5 (9.9-13.8) |
| p-value |  |  | 0.500 |

Abbreviations: PFS, progression free survival
