# Supplementary material for: Sequential RAS mutations evaluation in cell-free DNA of patients with tissue RAS wild-type metastatic colorectal cancer: the PERSEIDA (Cohort 2) study
Source: Clin Transl Oncol. 2024 Apr 20;26(10):2640–51. doi: 10.1007/s12094-024-03487-4 (PMC11410833; doi:10.1007/s12094-024-03487-4)
Supplement: Supplementary file 6 — (DOCX 19 KB) [file 12094_2024_3487_MOESM6_ESM.docx]

## Sequential *RAS* mutations evaluation in cell-free DNA of patients with tissue *RAS* wild-type metastatic colorectal cancer: The PERSEIDA (Cohort 2) Study

Clinical and Translational Oncology

## Manuel Valladares-Ayerbes, Maria José Safont, Encarnación González Flores, Pilar García-Alfonso, Enrique Aranda, Ana-Maria López Muñoz, Esther Falcó Ferrer, Luís Cirera Nogueras, Nuria Rodríguez-Salas, Jorge Aparicio, Marta Llanos Muñoz, Paola Patricia Pimentel Cáceres, Oscar Alfredo Castillo Trujillo, Rosario Vidal Tocino, Mercedes Salgado Fernández, Antonieta Salud-Salvia, Bartomeu Massuti Sureda, Rocio Garcia-Carbonero, Maria Ángeles Vicente Conesa, Ariadna Lloansí Vila, on behalf of the PERSEIDA investigators

Manuel Valladares Ayerbes

Hospital Universitario Virgen del Rocío, Instituto de Biomedicina, Sevilla, Spain

Email: [mvalaye@icloud.com](mailto:mvalaye@icloud.com)

## Table S4. PFS according to *RAS*, *BRAF*, and *RAS/BRAF* mutational status in liquid biopsy at baseline and any time (patients left-sided tumors, panitumumab subpopulation)

|  | **Wild-type** | **Mutant** | **Total** |
| --- | --- | --- | --- |
| ***At baseline*** |  |  |  |
| ***RAS*** | **n = 74** | **n = 3** | **n = 77** |
| PFS, % (95% CI) | 13.3 (11.0-15.9) | 9.7 (8.0-) | 13.0 (10.9-15.9) |
| p-value |  |  | 0.7558 |
| ***RAS* / *BRAF*** | **n = 73^a^** | **n = 4^b^** | **n = 77** |
| PFS, % (95% CI) | 13.3 (11.0-16.5) | 8.9 (2.4-) | 13.0 (10.9-15.9) |
| p-value |  |  | 0.736 |
| ***At any time*** | **Wild-type** (always) | **Mutant** (at any time) | **Total** |
| ***RAS*** | **n = 68** | **n = 9** | **n = 77** |
| PFS, % (95% CI) | 13.3 [10.9-15.9] | 13.0 [5.3-32.6] | 13.0 [10.9-15.9] |
| p-value |  |  | 0.710 |
| ***RAS* / *BRAF*** | **n = 67^a^** | **n = 10^b^** | **n = 77** |
| PFS, % (95% CI) | 13.3 (10.9- 15.9) | 11.3 (2.4 - 32.6) | 13.0 (10.9 - 15.97) |
| p-value |  |  | 0.990 |

Abbreviations: PFS, progression free survival.

^a^Patients with both *RAS* and *BRAF* wild-type always as per liquid biopsy at baseline.

^b^Patients with either *RAS* or *BRAF* mutant any time as per liquid biopsy at baseline.

^*^No statistical differences when 95% CI of odds ratio contains 1.
