# Supplementary material for: Sequential RAS mutations evaluation in cell-free DNA of patients with tissue RAS wild-type metastatic colorectal cancer: the PERSEIDA (Cohort 2) study
Source: Clin Transl Oncol. 2024 Apr 20;26(10):2640–51. doi: 10.1007/s12094-024-03487-4 (PMC11410833; doi:10.1007/s12094-024-03487-4)
Supplement: Supplementary file 7 — (DOCX 18 KB) [file 12094_2024_3487_MOESM7_ESM.docx]

## Sequential *RAS* mutations evaluation in cell-free DNA of patients with tissue *RAS* wild-type metastatic colorectal cancer: The PERSEIDA (Cohort 2) Study

Clinical and Translational Oncology

## Manuel Valladares-Ayerbes, Maria José Safont, Encarnación González Flores, Pilar García-Alfonso, Enrique Aranda, Ana-Maria López Muñoz, Esther Falcó Ferrer, Luís Cirera Nogueras, Nuria Rodríguez-Salas, Jorge Aparicio, Marta Llanos Muñoz, Paola Patricia Pimentel Cáceres, Oscar Alfredo Castillo Trujillo, Rosario Vidal Tocino, Mercedes Salgado Fernández, Antonieta Salud-Salvia, Bartomeu Massuti Sureda, Rocio Garcia-Carbonero, Maria Ángeles Vicente Conesa, Ariadna Lloansí Vila, on behalf of the PERSEIDA investigators

Manuel Valladares Ayerbes

Hospital Universitario Virgen del Rocío, Instituto de Biomedicina, Sevilla, Spain

Email: [mvalaye@icloud.com](mailto:mvalaye@icloud.com)

## Table S5. Univariable and multivariable linear regression model for tumor burden (Panitumumab subpopulation)

A. Univariable Analysis

| Effect |  | Estimate | Standard Error | t Value | Pr > \|t\| | Lower | Upper |
| --- | --- | --- | --- | --- | --- | --- | --- |
| Intercept |  | 16.73 | 43.40 | 0.39 | 0.70 | -69.46 | 102.92 |
| Liver metastasis | Yes | 41.45 | 11.19 | 3.70 | 0.0004 | 19.23 | 63.67 |
|  | No | Ref |  |  |  |  |  |
| *RAS* status baseline | Wild-type | 26.82 | 33.75 | 0.79 | 0.43 | -40.21 | 93.84 |
|  | Mutant | Ref |  |  |  |  |  |
| *BRAF* status baseline | Wild-type | -0.68 | 24.21 | -0.03 | 0.98 | -48.76 | 47.39 |
|  | Mutant | Ref |  |  |  |  |  |

Selection of the variables with a p value < 0.15 for the overall effect of the variable in the univariate linear regression model.

B. Multivariable Model

*Type 3 Tests of Fixed Effects*

| ***Effect*** | *F Value* | *Pr > F* |
| --- | --- | --- |
| Liver metastasis | 13.72 | **0.0004** |
| *RAS* status baseline | 0.63 | 0.43 |
| *BRAF* status baseline | 0.00 | 0.98 |

*Least Squares Means*

| *Effect* |  | *Estimate* | *Standard Error* | *t Value* | *Pr > \|t\|* | *Lower* | *Upper* |
| --- | --- | --- | --- | --- | --- | --- | --- |
| ***Liver metastasis*** | Yes | 71.25 | 20.37 | 3.50 | 0.0007 | 30.80 | 111.70 |
|  | No | 29.80 | 23.24 | 1.28 | 0.20 | -16.35 | 75.94 |

*Differences of Least Squares Means*

| *Effect* |  | *Estimate* | *Standard Error* | *t Value* | *Pr > \|t\|* | *Lower* | *Upper* |
| --- | --- | --- | --- | --- | --- | --- | --- |
| ***Liver metastasis*** | Yes vs No | 41.45 | 11.19 | 3.70 | 0.0004 | 19.23 | 63.67 |
