# Supplementary material for: Sequential RAS mutations evaluation in cell-free DNA of patients with tissue RAS wild-type metastatic colorectal cancer: the PERSEIDA (Cohort 2) study
Source: Clin Transl Oncol. 2024 Apr 20;26(10):2640–51. doi: 10.1007/s12094-024-03487-4 (PMC11410833; doi:10.1007/s12094-024-03487-4)
Supplement: Supplementary file 8 — (DOCX 20 KB) [file 12094_2024_3487_MOESM8_ESM.docx]

## Sequential *RAS* mutations evaluation in cell-free DNA of patients with tissue *RAS* wild-type metastatic colorectal cancer: The PERSEIDA (Cohort 2) Study

Clinical and Translational Oncology

## Manuel Valladares-Ayerbes, Maria José Safont, Encarnación González Flores, Pilar García-Alfonso, Enrique Aranda, Ana-Maria López Muñoz, Esther Falcó Ferrer, Luís Cirera Nogueras, Nuria Rodríguez-Salas, Jorge Aparicio, Marta Llanos Muñoz, Paola Patricia Pimentel Cáceres, Oscar Alfredo Castillo Trujillo, Rosario Vidal Tocino, Mercedes Salgado Fernández, Antonieta Salud-Salvia, Bartomeu Massuti Sureda, Rocio Garcia-Carbonero, Maria Ángeles Vicente Conesa, Ariadna Lloansí Vila, on behalf of the PERSEIDA investigators

Manuel Valladares Ayerbes

Hospital Universitario Virgen del Rocío, Instituto de Biomedicina, Sevilla, Spain

Email: [mvalaye@icloud.com](mailto:mvalaye@icloud.com)

## Table S6. Subclonal genomic variants detected by NGS technique

| Patient | Single nucleotide variant | | Copy Number Variant |
| --- | --- | --- | --- |
|  | **Variant** | **Gene** | **Gene** |
| 1 | Stop variant | *APC* (two variants) |  |
|  | Amino acid change | *TP53* |  |
| 2 | Stop variant | *APC* | *MET*  *EGFR* |
|  | Amino acid change | *BRAF* |  |
|  | Splicing variant | *FBXW7*  *TP53* |  |
| 3 | Amino acid change | *TP53* | *___* |
| 4 | Amino acid change | *JAK2*  *KRAS* (G13C) | ___ |
| 5 | Amino acid change | *BRAF*  *TP53* | *___* |
| 6 | Stop variant | *APC (two variants)*  *TP53* | *___* |
|  | Amino acid change | *FBXW7*  *PIK3CA* |  |
| 7 | Amino acid change | *KRAS* (A146T)  *TP53* | *MET*  *EGFR*  *ERBB2* |
|  | Splicing variant | *KDR* |  |
| 8 | Amino acid change | *EGFR*  *ERBB2*  *TP53* | *MET*  *EGFR*  *ERBB2* |
